# Income Inequality as a Moderator of the Relationship between Psychological Job Demands and Sickness Absence, in Particular in Men: An International Comparison of 23 Countries

**DOI:** 10.1371/journal.pone.0086845

**Published:** 2014-02-05

**Authors:** Johanna Muckenhuber, Nathalie Burkert, Franziska Großschädl, Wolfgang Freidl

**Affiliations:** Institute for Social Medicine and Epidemiology, Medical University Graz, Graz, Austria; University of Westminster, United Kingdom

## Abstract

**Objectives:**

The aim of this study was to investigate whether more sickness absence is reported in countries with higher income inequality than elsewhere, and whether the level of income inequality moderates the association between psycho-social job demands and sickness absence.

**Methods:**

Our analysis is based on the Fifth European Working Conditions Survey that compared 23 European countries. We performed multi-level regression analysis. On the macro-level of analysis we included the Gini-Index as measure of inequality. On the micro-level of analysis we followed the Karasek-Theorell model and included three scales for psychological job demands, physical job demands, and decision latitude in the model. The model was stratified by sex.

**Results:**

We found that, in countries with high income inequality, workers report significantly more sickness absence than workers in countries with low income inequality. In addition we found that the level of income inequality moderates the relationship between psychological job demands and sickness absence. High psychological job demands are significantly more strongly related to more days of sickness absence in countries with low income inequality than in countries with high income inequality.

**Conclusions:**

As the nature and causal pathways of cross-level interaction effects still cannot be fully explained, we argue that future research should aim to explore such causal pathways. In accordance with WHO recommendations we argue that inequalities should be reduced. In addition we state that, particularly in countries with low levels of income inequality, policies should aim to reduce psychological job demands.

## Introduction

The purpose of this study was to analyze whether psychological demands at the workplace differ in their association with the number of days of sickness absence when comparing countries with high income inequality to countries with low income inequality.

There is an ongoing debate about whether income inequality is detrimental to health or not [1]. Wilkinson et al. argue that more unequal societies have lower average standards of health and shorter life expectancy [2] and there is empirical evidence to support Wilkinson's theory [3]–[6]. Waldmann, for example, found higher infant mortality rates in countries with higher income inequality in comparison to those with lower income inequality, despite the fact that the countries had comparable levels of low income [7]. Muntaner and Lynch argue that Wilkinson's approach to focus on income inequality as a determinant of population health is very important for understanding inequalities in health [8]. Studies using the Partial Concentration Index (an index similar to the Gini Index) have shown that inequality in health increased over time as a result of increasing income inequality and a higher average income [9].

There is, however, also contradictory evidence. A number of studies argue that Wilkinson's theory about the impact of relative inequality on health represents an artifact due to the use of aggregate data and that this artifact is due to a curvilinear relation between income and health [10]. Karlsson et al. report that the association between total income and health is not totally linear but curvilinear and that the degree of curvilinearity differs between countries [1]. Some studies show that low income is detrimental to health, but that Wilkinson's hypothesis cannot be supported since no significant relationship between health and income inequality was found [11]. Karlsson et al. report only minor evidence supporting the income inequality hypothesis and state that, in particular within the group of high income countries, individuals with a low income report better average health in countries with less marked inequality than in countries with strong inequality. (Karlsson et al. 2010)

Psychological and physical demands at the workplace are known to be detrimental to health [12]–[14]. Karasek and Theorell, in their demand/control model, argue that psychological and physical job demands have negative effects on health, but that decision latitude is positively related to health [15]. Other research found psychosocial job demands to be related to psychological distress [16]–[18]. In addition, job control has been found to be an important factor when it comes to maintaining psychological well-being [13], [18]. One aspect of the demand-control model relates to the concept of job strain, which is defined as a combination of high job demands and low decision latitude. Research only partly supports the hypothesis that a high decision latitude buffers the negative effects of job demands on health [13], [19].

Health, wellbeing, and mortality are all strongly related to the number of days of sickness absence [20], [21], and are reported to show a stable pattern over the years [22]. Research on work and health showed high job demands to be associated with a higher number of days of sickness absence [21].

Therefore we argue that sickness absence is an important outcome variable for the analysis of the relationship between job demands and health, in particular when accounting for country-by-country differences in the level of income inequality.

To our knowledge this is the first ever study to examine whether the effects of job demands on the number of days taken of sickness absence differ between countries with high income inequality, and those with low income inequality.

## Methods

### Data

The database for this article is the Fifth European Working Conditions Survey (EWCS) which is open accessible to researchers [23]. The data were anonymized directly during the process of data collection. The study was carried out in compliance with the declaration of Helsinki. The ethics committee of the Medical University of Graz approved this study. The survey was carried out in 2010 by the European Foundation for the Improvement of Living and Working Conditions [24]. Gallup Europe conducted a cross-sectional survey comprising a multi-stage random sample of workers who were interviewed using a face-to-face questionnaire. The target number per country was 1,000 persons. The data base of this article comprised a total of 30,089 workers in 23 countries (Albania, Austria, Belgium, Bulgaria, Croatia, Estonia, Finland, FYROM, Germany, Greece, Hungary, Ireland, Italy, Latvia, Lithuania, Montenegro, Norway, Poland, Romania, Slovenia, Spain, Sweden, Turkey).

### Measures

#### Dependent Variable

**Sickness absence:** “Over the past 12 months, how many days in total were you absent from work for reasons of health problems?” (A metric variable starting with 0.)

#### Independent Variables

**Macro-level of analysis: Gini Index:** We used the Gini Index as macro (aggregate)-level variable. The Gini Index is a common measure of income inequality and its relation to health (1,2,8). It is a “measure of the deviation of the distribution of income among individuals within a country from a perfectly equal distribution. A value of 0 represents absolute equality, a value of 100 absolute inequality.” [25]
**Micro-level (individual level) of analysis:** We analyzed associations between sickness absence and work demands and controlled for the socio-economic status and the socio-demographic status.
**Variables of the demand/control model by Karasek and Theorell:** We measured job demands on the basis of three factors of the demand/control model by Karasek and Theorell. To achieve this, we constructed three indices (psychological demands, physical demands, and decision latitude). We followed an approximate approach using variables of the EWCS, since this data set contained none of the original values from the Job Control Questionnaire (JCQ). We tested the three indices by means of factor analysis and standardized them into ranges with a lowest possible value of 0 and a highest possible value of 100.
  The index “psychological job demands” consists of 10 variables (Alpha = 0.62, 100 = high psychological demands). The scale includes the following items: “Does your job involve … –short repetitive tasks of less than 1 minute?”; “… – short repetitive tasks of less than 10 minutes?”; “… – working at very high speed?”; “… – working to tight deadlines?”; “Generally, does your main paid job involve monotonous tasks?”; “Select the response which best describes your work situation: a) Your job gives you the feeling of work well done; b) You have the feeling of doing useful work; c) You know what is expected of you at work; d) Your job involves tasks that are in conflict with your personal values; e) You experience stress at work.”
  The index “physical job demands” includes 14 variables (Alpha = 0.83, 100 = high physical demands at work). The scale comprises the following items: “*Are you exposed at work to* - Vibrations from hand tools, machinery, etc.?”; “… – Noise so loud that you would have to raise your voice to talk to people?”; “… – High temperatures which make you perspire even when not working?”; “… – Low temperatures whether indoors or outdoors?”; “… – Breathing in smoke, fumes, powder or dust etc.?”; “… – Breathing in vapors such as solvents and thinners?”; “… – Handling or being in skin contact with chemical products or substances?”; “… – Tobacco smoke from other people?”; “… – Handling or being in direct contact with materials which can be infectious, such as waste, bodily fluids, laboratory materials, etc.?”; “*Does your main paid job involve* – Tiring or painful positions”; “… – Lifting or moving people”; “… –- Carrying or moving heavy loads”; “… – Standing”; “… – Repetitive hand or arm movements”.
  The third index “decision latitude” consists of 8 items (Alpha = 0.61, 100 = a high level of decision latitude). It includes the following items: “On the whole, is your pace of work dependent, or not: a) on the work done by colleagues?; b) on numerical production targets or performance targets?”; “Are you able to choose or change: a) your order of tasks?; b) your methods of work?; c) your speed or rate of work?”; “Select the response which best describes your work situation: a) You can take a break when you wish; b) You are able to apply your own ideas in your work; c) You can influence decisions that are important for your work.”
**Socio-economic and socio-demographic status (SEDS):** We measured socio-economic status and socio-demographic status based on:
  - Education: “What is your highest level of education?” With the categories “primary level (1)”, “low secondary”, “high secondary” and “tertiary (4)”.
  - Age in years
  - Subjective perception of income: “Thinking of your household's total monthly income, is your household able to make ends meet?” With the categories “Very easily (1)”, “easily”, “fairly easily”, “with some difficulty”, “with difficulty”, “with great difficulty (6)”.

### Statistical Analysis

We stratified the analysis by sex and calculated the models separately for men and women in order to investigate gender differences. We performed multi-level analyses which, as a kind of comprehensive analysis, are also considered to be suitable for this purpose [26].

We calculated multi-level regression models in order to ensure a comparison between the countries and accounting for the structure of the data with one macro-level variable (the Gini Index) and the individual level of the other variables. As we were interested to know whether the Gini Index influences the relationship between work demands and number of days of sickness absence, we investigated cross-level interaction effects using the Gini Index as macro–variable, and psychological job demands and sickness absence as micro-variables. In addition we calculated an interaction term between psychological demands and decision latitude at the micro-level of analysis.

We considered the hierarchical structure of the data and controlled for the level of the independent variables. To this end, we used the SPSS Mixed Model procedure and calculated linear random intercept and random slope multilevel regression models using REML (Residual Maximum Likelihood Estimation) as the estimation type. On a nominal level we used the country identification variable as subject identification. We compared countries by their value in the Gini Index as metric macro-level variable.

We used centered variables in order to avoid problems of multi-collinearity which can occur when calculating interaction effects [27]. The following formula was used to center the variables: X_i_-Mean Score of X.

In addition we calculated analyses of variance (ANOVA) in order to determine bivariate differences between countries with a high, a medium, and a low Gini Index regarding the level of psychosocial work demands.

## Results

### Descriptive Overview

52% of the sample were men. For days taken of sickness absence, the survey yielded a mean value of 5.40 (SD = 18.34) days (men) and 6,59 (SD = 20.50) days (women). The mean age of men was 41.58 (SD = 12.39) years, that of women 41.79 years (SD = 11.90). 7.3% of men and 5.1% of women had primary education, 20.1% of men and 18.3% of women low secondary, 46.3% of men and 43.7% of women secondary, and 26.3% of men and 32.9% of women had tertiary education.

The mean value obtained in the subjective perception of income among men was 3.30 (SD = 1.33) and among women 3.37 (SD = 1.32). The means for psychological demands, physical demands, and decision latitude amounted to 21.19 (SD = 15.95) in men and 29.47 (16.05) in women; to 23.61 (SD = 17.31) in men and 17.38 (13.07) in women; and to 42.61 (SD = 19.43) in men and 41.45 (18.90) in women respectively.

Finally the mean Gini Index across the whole sample was 33.56, within a range from 25 to 45 (SD = 4.76).

#### Associations between Gini Index and psychosocial work demands

Analyses of variance showed that men and women experience significantly more psychological work demands in countries with low income inequality in comparison to countries with a high Gini Index (men: df = 2, p = 0.000; women: df = 2, p = 0.039).

A converse effect can be shown for physical demands. Both men and women reported more physical demands in countries with high income inequality when compared to countries with low income inequality (men: df = 2, p = 0.000; women: df = 2, p = 0.000).

Regarding decision latitude, we found women in more egalitarian countries to report higher levels of decision latitude than those living in countries with high income inequality (df = 2, p = 0.002). No significant difference was found for the men.

### Multilevel- multivariate Results

Table 1 shows the results of the multilevel regression model. The various Coefficients B with a Confidence Interval of 95% are shown for men and women separately. The higher the value for the Regression Coefficient B, the stronger the influence of the variable in question.

**Table 1:** Multilevel analysis: dependent variable = sickness absence.

	male	female
	Regression Coefficient B (95% CI min/max)	Regression Coefficient B (95% CI min/max)
Intercept	−1,261(−3,086–0,56)4	0,765(−1,349–2,879)
Subjective perception of income (1 = very good, 6 = very bad)	0,643(0,361–0,925)***	0,463(0,131–0,795)**
[Education = primary]	2,305(0,733–3,876)**	−0,787(−2,935–1,361)
[Education = 2.00]	1,632(0,550–2,714)**	0,083(−1,172–1,339)
[Education = 3.00]	0,052(−0,801–0,905)	−0,645(−1,549–0,259)
[Education = tertiary]	Category of reference	Category of reference
Age	0,092(0,064–0,119)***	0,106(0,073–0,140)***
Psychological demands	0,035(0,002–0,067)*	0,050(0,012–0,087)**
Physical demands	0,076(0,028–0,124)**	0,114(0,067–0,160)***
Decision latitude	−0,027(−0,054–−0,001)*	−0,043(−0,079–−0,007)**
Gini-Index	−0,219(−0,431–−0,008)*	−0,290(−0,515–−0,065)**
Cross-Level Interaction Effects		
Psychological demands*HDI	−0,008(−0,015–−0,002)*	−0,009(−0,016–−0,001)**
Physical demands*HDI	−0,005(−0,014–0,005)	−0,009(−0,018–0,000)
Decision latitude*HDI	0,003(−0,003–0,008)	0,001(−0,006–0,008)
Decision latitude *psychological demands	0,000(−0,001–0,001)	−0,001(−0,002–0,001)

Significance:

* p = 0.01–0.05,

** p = 0.001–0.009,

*** p<0.001.

### Gini Index and Sickness Absence

The multi-level analysis shows that higher income inequality is associated with more days of sickness absence even when controlling for socio-economic status and socio-demographic status. A comparison between the Coefficient B and the Confidence Intervals has shown this association to be even stronger in women than in men.

### Psychosocial Work Demands and Sickness Absence

In both men and women, higher psychological demands and higher physical demands are significantly associated with more days of sickness absence. By contrast, a greater decision latitude is significantly related to fewer days of sickness absence.

We found no significant interaction effect between psychological demands and decision latitude.

### Socio-Economic and Socio-Demographic Status and Sickness Absence

A higher subjectively perceived income was found to be significantly associated with less sickness absence.

Lower education is significantly related to more sickness absence, but only in men. Among women, no significant association between education and sickness absence was found.

Higher age is associated with higher levels of sickness absence in both men and women.

### Cross-Level Interaction Effects between Gini Index, Psychosocial Work Demands and Sickness Absence

**Figure 1:**
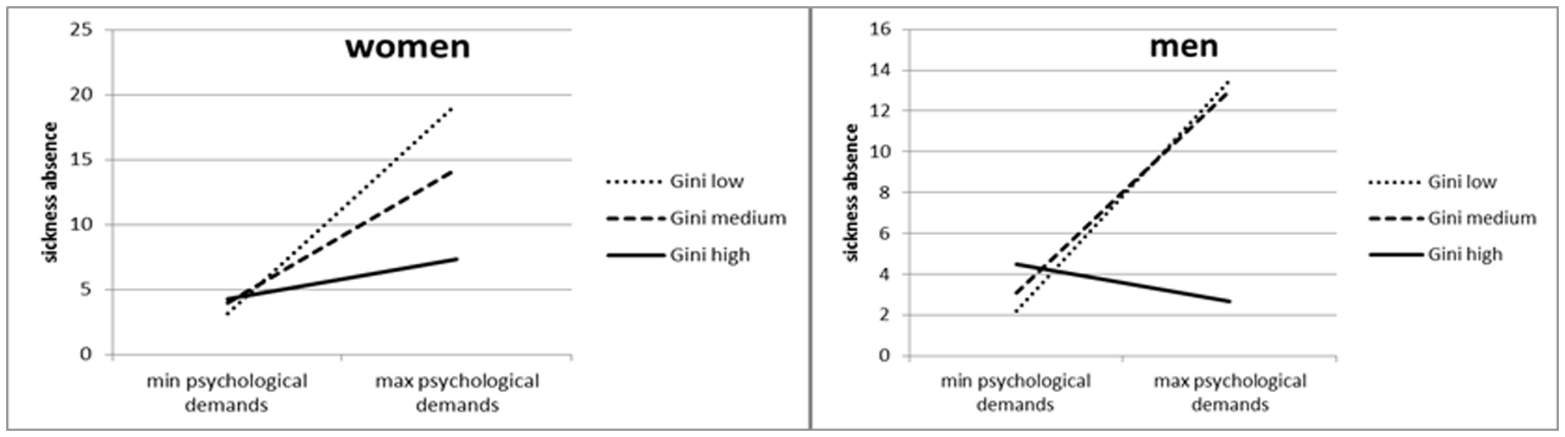
Cross-level interactions between psychological demands and sickness absence in countries with low, medium, and high Gini Indices.

Table 1 and Figure 1 show cross-level interaction effects between the Gini Index and psycho-social work demands in their associations with sickness absence. The interaction lines are presented for tertiles of the Gini Index and differentiate between low (25–30), medium (30.1–34.5), and high (34.7 through 46). The following formula was used in order to calculate the predicted values (y) of days in sickness absence, as shown in the graphs.

Y = [B(psychological demands (for the respective Gini Index group)*psychological demands+d ( = Intercept)].

The regression coefficients of the linear regression models for each respective Gini Index group were used as B values.

For both women and men, significant cross-level interaction effects were found, with the level of income inequality moderating the association between psychological job demands and sickness absence. The strongest link between the levels of psychological demands and sickness absence was found in countries with a low Gini Index (with low income inequality). Figure 1 presents these findings using steep regression lines. Similarly for both men and women, this association points in the same direction but is less marked in countries with a medium Gini Index.

No significant cross-level interaction effects were found between income inequality and physical demands or between income inequality and decision latitude.

### Gender Differences in the Association Between Psychological Job Demands and Sickness Absence in Countries with High Income Inequality

In countries with high income inequality, men and women demonstrated a different association between psychological job demands and sickness absence. Within these countries, we found a weakly positive association between psychological job demands and sickness absence for women. However, Figure 1 also shows that, among men, higher psychological demands are associated with fewer sickness absence days.

## Discussion

This study presents new findings and contributes to advances in the field, since it is the first ever study to our knowledge to analyze cross-level interactions with a view to determining whether the level of income inequality in a country moderates the relationship between psychosocial work demands and sickness absence.

Previous research did find country differences in the prevalence of poor wellbeing and other health-related items, but also in the prevalence of psychosocial work demands and in subjective health [28], [29]. Former cross-country comparisons also revealed that the link between wealth and health is stronger in countries with a more unequal distribution of economic resources [30].

We measured income inequality on the macro-level using the Gini Index. In line with previous research [3], [4], [7], our analysis shows that a higher level of income inequality is related to poorer health, in this study measured by the proxy of a higher number of days of sickness absence. Therefore we argue that our study supports Wilkinson's theory about the effects of income inequality on health.

We found significant differences in the level of psychosocial work demands depending on the level of income inequality. In countries with low income inequality, both men and women reported more psychological job demands when compared to workers in high income inequality countries. A converse effect could be shown regarding physical job demands. We argue that higher levels of physical work demands might be due to poorer working conditions in countries with high income inequality as opposed to those in low-income-inequality countries. Moreover we argue that individuals experiencing high levels of physical work demands might be focused on these and might thus be less attentive to psychological work demands. In workers in low-income-inequality countries we might find the reverse effect. As workers in these countries experience lower levels of physical work demands they might be more alert to psychological work demands.

As a consequence, we argue that in countries with high income inequality, preventive activities should focus on improving the physical conditions at the workplace. In low Income-inequality countries preventive activities should focus on psychological conditions at the workplace.

We found women to be more likely than men to take sickness absence. This effect was stronger in more egalitarian countries. We argue that women might be more stressed than men by the double burden of work in their job and of the reproductive work they have to accomplish. This might be a reason for a poorer state of health among women and hence for the necessity to take more days of sickness absence. Since in countries with higher income inequality the fear of losing the job might be stronger than in more egalitarian countries, women in countries with high income inequality might be reluctant to stay at home, even when ill. In addition, it seems highly possible particularly in more egalitarian countries that women are more willing to show preventive behavior than men. In this context we argue that an additional explanation for gender differences might be that women are more likely than men to take sickness absence when experiencing health problems.In line with Karasek's hypothesis and with previous research [29], though leaving cross-level interactions aside, we found stronger psychological demands and stronger physical demands but less decision latitude to be associated with more sickness absence. This applies to both men and women.

The cross-level interaction shows that the level of income inequality moderates this association. The relation between a high level of psychological demands and a great number of days of sickness absence is strongest in countries with low income inequality. For women, it can be shown that in countries with high income inequality, the association between psychological job demands and sickness absence points in the same direction, but is less marked.

Research [16]–[18] has shown psychological job demands to be associated with illness, psychological symptoms, and mental health problems. Therefore it is likely that individuals who experience strong psychological demands need more days of rest in the form of sickness absence than their colleagues who experience fewer psychological demands.

In order to explain the differences between countries with low and high income inequality, we argue that in countries with high income inequality, psychological health problems are possibly less accepted as illness than in low income inequality countries. In the former setting it is likely that individuals are more reluctant to go on sick leave even though they experience strong psychological demands at work.

This greater willingness to recognize psychological health problems might also have a corresponding legal basis in low inequality countries where their laws make it easier to take sick leave because of mental health problems.

For men, the association between psychological demands and sickness absence is actually reversed in countries with high income inequality, in contrast to countries with low income inequality. In these countries, men reporting strong psychological demands have fewer days of sickness absence than men reporting weak psychological demands.

We argue that this gender difference, with the moderation effect of income inequality being particularly strong in men, could be partly due to cultural differences between high-inequality and low- inequality countries. It might be even less acceptable for men than for women to have mental health problems, in particular in high-inequality countries. As a consequence, men might experience even more pressure than women and might thus be more reluctant to stay at home, despite their problems, out of fear of losing their job or having difficulties to find a new job.

### Strengths and Limitations

An important strength of this study is the use of the Gini Index as a metric macro-level variable in order to show cross-level interaction effects between the Gini Index and psycho-social work demands.

One limitation of our study concerns the interpretation of cross-level interaction effects. Further research will be required for a more in-depth interpretation of these effects. Another limitation concerns the measurement of the dimensions of Karasek's and Theorell's model. Since the EWCS questionnaire did not include the original items of the scale, we had to use proxies. Our factor analyses supports the construction of the scales. In addition we contend that this is a legitimate approach, as previous research [21], [31] has proven EWCS data to be a sound basis for constructing scales following the Karasek-Theorell model.

Another strength of this study is the use of a large cross-national data set, which allows comparisons between countries with different levels of income inequality.

Causal conclusions are, however, impossible due to the cross-sectional nature of the data. Assumptions regarding the direction of associations can only be put forward based on the underlying theory and on previous research.

### Conclusions

To sum up, we argue that the nature and causal pathways of cross-level interaction effects still cannot be fully explained, hence future research should aim to explore such causal pathways.

Our findings confirm that a high level of psychological job demands seems to be most strongly associated with more sickness absence in low-inequality countries. Therefore preventive action in low-inequality countries should in particular aim to improve the psychological working environment.

In addition, we found that high income inequality is associated with higher levels of sickness absence. Therefore we argue, in line with WHO recommendations [32], that in order to improve the health status of the working population but also in order to ensure their ability to work, inequality should be reduced.
